# TO901317 regulating apolipoprotein M expression mediates via the farnesoid X receptor pathway in Caco-2 cells

**DOI:** 10.1186/1476-511X-10-199

**Published:** 2011-11-04

**Authors:** Chunhua Zhu, Dongmei Di, Xiaoying Zhang, Guanghua Luo, Zongchun Wang, Jiang Wei, Yuanping Shi, Maria Berggren-Söderlund, Peter Nilsson-Ehle, Ning Xu

**Affiliations:** 1 Department of Cardiothoracic Surgery, Third Affiliated Hospital of Suzhou University, Changzhou 213003, P.R. China; 2 Comprehensive Laboratory, Third Affiliated Hospital of Suzhou University, Changzhou 213003, P.R. China; 3 Division of Clinical Chemistry and Pharmacology, Department of Laboratory Medicine, Lunds University, S-221 85 Lund, Sweden

**Keywords:** Liver X Receptor, Farnesoid X Receptor, Caco-2 cell line, Apolipoprotein M

## Abstract

**Background:**

Apolipoprotein M (apoM) may have potential antiatherosclerotic properties. It has been reported that apoM expression could be regulated by many intracellar and extracellar factors. In the present study we further investigated regulation of apoM expression in Caco-2 cells stimulated by a liver X receptor (LXR) agonist, TO901317.

**Materials and methods:**

Caco-2 cells were cultured in the presence of either TO901317, farnesoid X receptor (FXR) antagonist guggulsterone or TO901317 together with guggulsterone at different concentrations for 24 hrs. The mRNA levels of ATP-binding cassette transporter A1 (ABCA1), apoA1, apoM, liver receptor homologue-1 (LRH-1) and short heterodimer partner 1 (SHP1) were determined by real-time RT-PCR.

**Results:**

When Caco-2 cell cultured with TO901317 alone, the mRNA levels of ABCA1, apoA1, apoM, LRH-1 and SHP1 were significantly increased with dose-dependent manners (*p *< 0.05), whereas when the cells cultured with guggulsterone alone, the mRNA levels of apoM, SHP1 and LRH-1 (*p *< 0.05) were strongly inhibited. Moreover, guggulsterone could abolish the TO901317 enhanced mRNA levels of apoA1 apoM, SHP1 and LRH-1.

**Conclusion:**

The present study demonstrated that LXR agonist TO901317 induced apoM expression in Caco-2 cells might be mediated via the LXR/FXR pathway.

## Introduction

With the aging population and changing lifestyles, the incidence of cardiovascular diseases (CVD) has gradually increased [1]. Abnormal lipid metabolism has been considered as one of the major risk factors of CVD [2]. Previously studies have demonstrated that serum concentrations of apolipoprotein (apo) AI and apoB have significantly correlation with the occurrences of CVD [3,4], and other apolipoproteins may also involve in the initiation and progression of the diseases [5]. ApoM is one of the latest discovered apolipoproteins that is mainly synthesized in the liver, and to a smaller amounts, in the kidney [6]. In human plasma, most apoM are located in the high-density lipoproteins (HDL) and small proportion present also in apoB-containing lipoproteins, i.e. chylomicrons, very low-, and low-density lipoproteins (VLDL and LDL) [6,7]. Recent investigations have suggested that apoM may participate in the HDL-related biological activities as an important component of HDL particle on the protection of endothelial cells [8]. Wolfrum, et al., [9] reported that apoM is required for preβ-HDL formation and cholesterol efflux to HDL particles, which is an initial and crucial stage of reverse cholesterol transport, and subsequently protects against atherosclerosis. In addition, the physiological and patho-physiological roles of apoM may also involve in the inflammatory activities and the potential immuno- and inflamm-reactive properties, and apoM may therefore contribute to the anti-inflammatory function of HDL, being as generally acknowledged as a significant antiatherogenic mechanism [10,11].

ApoM could be regulated by many factors including leptin, insulin, hyperglycemia and many cytokines *in vivo *and *in vitro *[12]. It has been demonstrated that apoM gene expression could be also affected by some nuclear receptors, such as hepatocyte nuclear factor-1α (HNF-1α) [13], hepatocyte nuclear factor-4α (HNF-4α) [12] and liver receptor homolog-1 (LRH-1) [12]. Liver X receptor (LXR) is a nuclear receptor, as a lipid sensor, protects cells from lipid overload and directly or indirectly controls apolipoprotein-mediated cholesterol efflux [14]. Our previous studies demonstrated that the synthetic LXR agonist TO901317 could down-regulate hepatic apoM expression *in vivo *and *in vitro *[15]. Whereas Calayir., et al. [16], in recognition of our findings that TO901317 inhibited apoM expression in HepG2 cells, even found that TO901317 could upregulate apoM expression in intestinal cells. In the present study we further revealed the regulative pathway of apoM expression in Caco-2 cells stimulated by TO901317.

## Materials and methods

### Cells and reagents

Human colorectal adenocarcinoma cell line, Caco-2, was obtained from the American Type Culture Collection (ATCC, Manassas, VA, USA). TO901317 was purchased from the Cayman Chemical Company (Ann Arbor, MI, USA). Guggulsterone was from the Sigma Chemical Co. Ltd. (Shanghai, China). Six-well cell culture clusters and 75 cm^2 ^vented cell culture flasks were purchased from the Nunc (Roskilde, Denmark). Fetal bovine serum (FBS) and Dulbecco's Modified Eagle Medium (DMEM) were obtained from the Invitrogen (Shanghai, China). Total RNA purification kits were purchased from the Shenergy Biocolor BioScience and Technology Company (Shanghai, China). First strand cDNA synthesis kits were obtained from the Fermantas (Vilnius, Lithuania). The LightCycler real-time RT-PCR System was from the Roche Applied Science (Mannheim, Germany).

### Cell cultures

Caco-2 cells were cultured in DMEM supplemented with 20% FBS in the presence of 100 U/ml penicillin, 100 μg/ml streptomycin and 1% Glutamax at 37°C under 5% CO_2 _atmosphere. Cells were plated in 6-well cell culture clusters at a density of 3 × 10^5 ^cells/dish with DMEM containing 20% FBS. Cell monolayer of approximately 50-70% confluence were grown for 24 hrs in the above media prior to experiments, cells were washed twice with phosphate buffered saline (PBS) and once with serum-free DMEM without antibiotics. Then the experimental medium, containing DMEM with 1% bovine serum albumin (BSA) and different concentrations of TO901317 and guggulsterone, or TO901317 together with guggulsterone were added. As TO901317 must be dissolved in the DMSO and then added into the experimental medium (the final concentration of DMSO was at 1% in the present study), 1% DMSO were always applied in the controls. Unless stated otherwise, cells were incubated at 37°C for 24 hrs.

### Total RNA extraction and real time RT-PCR

Total RNA of Caco-2 cells were extracted by using the total RNA purification kit according to the manufacturer's instructions. Primer Express software (Applied Biosystems) was applied to design the primers and probes of human apoM, apoA1, SHP1, LRH-1, ABCA1 and GAPDH (the sequences of primers and probes are shown in Table 1). Quantifications of mRNA levels were performed on a LightCycler in a final volume of 25 μl. Optimal conditions were obtained with 2.5 μl of 10 × PCR buffer, 1.5 μl of 25 mM MgCl_2_, 0.5 μl of 10 mM 4 × dNTPs, 0.25 μl of 5 U/μl Taq DNA polymerase, 0.1 μl of 100 μM specific sense primer, 0.1 μl of 100 μM specific antisense primer, 0.1 μl of 100 μM specific probe and 2 μl template cDNA. Finally 17.95 μl H_2_O was added to the reaction mixture. The thermal cycling conditions for human apoM, apoA1, SHP1, LRH-1, ABCA1 and GAPDH were as the following steps: 25°C for 10 min, 48°C for 30 min and 95°C for 5 min to do reverse transcription, and then the reaction mixture was preheated for 2 min at 50°C and for 10 min at 95°C to activate Taq polymerase. After that, a 40-cycle two step PCR was performed consisting of 15s at 95°C and 1 min at 60°C. Samples were amplified simultaneously in triplicates in one-assay run. The prospective amplicon of each gene was amplified and purified, then ligated into the pMD19-T vector. The ligated product was transformed into the E. Coli JM109 competent cells. In brief, a serial dilution of extracted plasmid DNA was used to generate a standard curve by plotting the cycle threshold versus the log initial copy number of input plasmid DNA. Standard curves of apoM, apoA1, SHP1, LRH-1, ABCA1 and GAPDH achieved a very high correlation coefficient (r = 1.00). The ratio between the target gene and GAPDH was calculated as the relative gene expression.

**Table 1:** Primers and fluorescent probes for real-time RT-PCR

Gene	Forward primer	Reverse primer	Probe
**GAPDH**	ggaaggtgaaggtcggagtc	cgttctcagccttgacggt	5'-FAM-tttggtcgtattgggcgcctg-TAMRA
**ApoM**	tgccccggaaatggatcta	cagggcggccttcagtt	5'-FAM-cacctgactgaagggagcacagatctca-TAMRA
**ApoA1**	ctgggataacctggaaaaggagac	ggaagtcgtccaggtagggct	5'-FAM-agatgagcaaggatctggaggaggtgaa-TAMRA
**SHP1**	aaagggaccatcctcttcaacc	agggttccaggacttcacacag	5'-FAM-cctccaagccgcctcccacatt-TAMRA
**LRH-1**	ttagtggcaaaacttcgttctctcc	agggcggcattgacttgttc	5'-FAM-agttcgtatgtctgaaattcttggtgctct-TAMRA
**ABCA1**	caaggggtaggagaaagagacgc	ctcagccagcacccccag	5'-FAM-ccagccacggcgtccctgctgt-TAMRA

### Statistical analysis

Data are expressed as means ± SE. All experiments were repeated twice and the data were represented one of the experiments. Statistical analyses were performed with the software Prism (version 5.0). Multiple comparisons were performed with one-way ANOVA/dunnett-t, and comparisons between two groups were statistically evaluated by the unpaired t-test. Significance was established at a *P *value less than 0.05.

## Results

**Figure 1:**
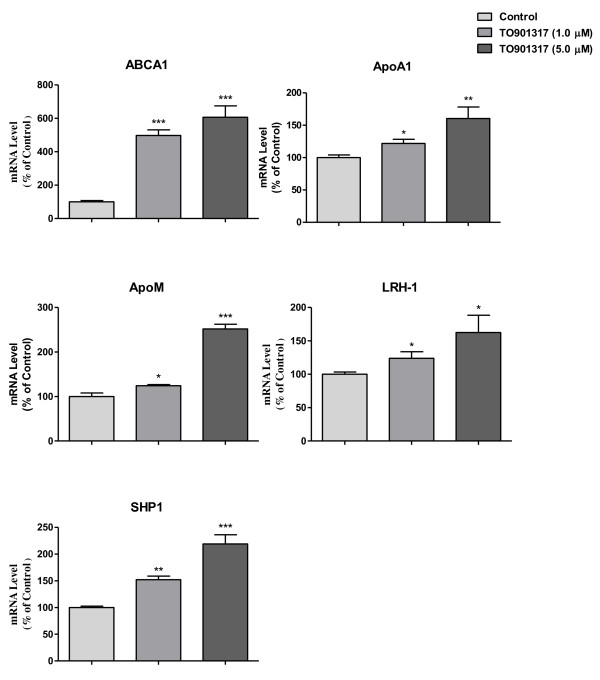
**Effects of TO901317 on mRNA levels of ABCA1, apoA1, apoM, LRH-1 and SHP1 in Caco-2 cells**. Caco-2 cells were cultured with different concentrations of TO901317 for 24 hrs. The mRNA levels of ABCA1, apoA1, apoM, LRH-1 and SHP1 were determined by real-time RT-PCR in triplicates. The controls were represented as 100%. Data represents one of two similar experiments (means ± SE, n = 5). *p < 0.05; **p < 0.01; ***p <0.001 vs. controls.

**Figure 2:**
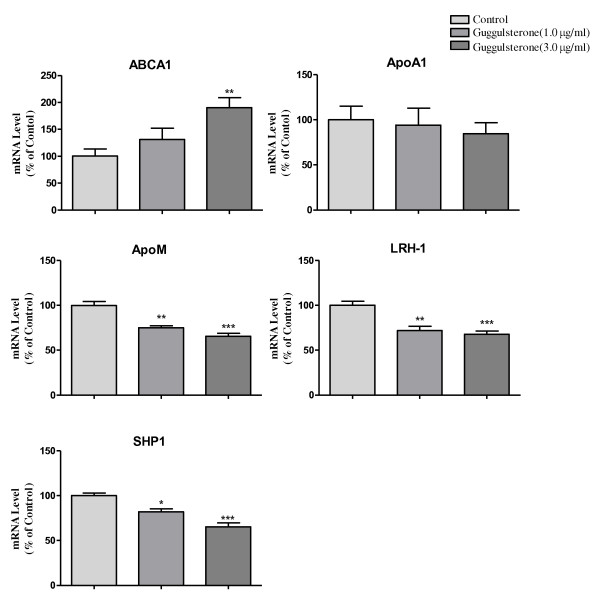
**Effects of guggulsterone on mRNA levels of SHP1, LRH-1 and apoM in Caco-2 cells**. Caco-2 cells were cultured with different concentrations of guggulsterone for 24 hrs. The mRNA levels of ABCA1, apoA1, SHP1, LRH-1 and apoM were determined by real-time RT-PCR in triplicates. The controls were represented as 100%. Data represents one of two similar experiments (means ± SE, n = 4-6). *p < 0.05; **p <0.01; ***p <0.001 vs. controls.

**Figure 3:**
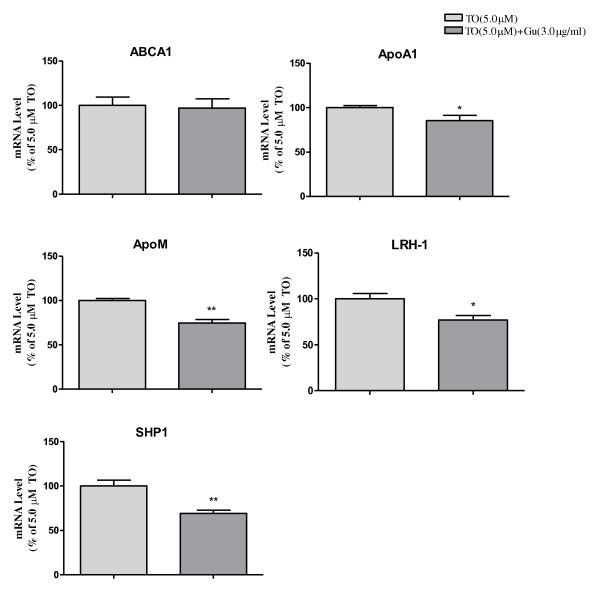
**Inhibitory effects of guggulsterone on TO901317 induced expressions of SHP1, LRH-1 and apoM in Caco-2 cells**. Caco-2 cells were cultured with 5 μM TO901317 together with 3.0 μg/ml guggulsterone for 24 hrs. The mRNA levels of ABCA1, apoA1, SHP1, LRH-1 and apoM were determined by real-time RT-PCR in triplicates. The cells cultured with TO901317 alone were represented as 100%. Data represents one of two similar experiments (means ± SE, n = 4-6). *p < 0.05; **p <0.01; ***p <0.001 vs. controls.

As shown in Figure 1, TO901317 could significantly upregulate mRNA levels of ABCA1, apoA1, apoM, LRH-1 and SHP1 with dose-dependent manners. At 1.0 μM TO901317, apoM mRNA level was increased by 24% (*P *< 0.05), and at 5.0 μM TO901317, increased by more than 100% (*P *< 0.01) compared to the controls. When Caco-2 cells were cultured with guggulsterone alone, mRNA levels of SHP1, LRH-1 and apoM were significantly inhibited (*P *< 0.05); apoA1 decrease by 6% and 16% respectively; However, ABCA1 were significant upregulated (Figure 2), which were also dose dependents. As shown in Figure 3, it demonstrated that guggulsterone could abolish TO901317 induced upregulation of apoA1, SHP1, LRH-1 and apoM. When Caco-2 cells cultured with 5 μM TO901317 together with 3.0 μg/ml guggulsterone, the mRNA levels of apoA1, SHP1, LRH-1 and apoM were only 85%, 70%, 77% and 74% compared to the cells cultured with TO901317 alone, respectively, whereas ABCA1 were not affected compared to the cells cultured with TO901317 alone (Figure 3).

## Discussion

ApoM has firstly been identified from human postprandial lipoproteins by Xu and Dahlbäck in 1999 [6]. Recent observations strongly suggest that apoM is predominantly confined to the HDL particles in human plasma and it may have antiatherogenic properties involving in the conversion of large HDL to pre-β HDL, the later mainly functions as an acceptor of peripherally deposited cholesterol that is described as the revise cholesterol transportation [9,17]. Moreover other researches indicated that apoM might be also involved in the immunity, inflammation, and neoplasia [18-20]. However, the physio-pathological functions of apoM are still not fully revealed. Observation of the regulation of apoM expression may reveal clinical importance of apoM in humans.

Previous studies have demonstrated that hepatic apoM expression could be regulated by certain cytokines and nuclear factors. In our previous study [15], we demonstrated that LXR agonist, TO901317, could significantly downregulate apoM expression in the hepatic cell line, HepG2 cells. Moreover, Calayir., et al. [16] confirmed our findings that TO901317 did inhibit apoM expression in HepG2 cells, however, they found that TO901317 could significantly upregulate apoM expression in intestinal cell, which suggest that TO901317 regulating apoM expression is cellular dependent. Moreover, Mosialou., et al. [12] reported that the natural LXR ligand oxysterols could significantly upregulate apoM mRNA level and protein level in HepG2 cells, and LXR could bind to the HRE in the proximal apoM promoter examined by the Chromatin Immunoprecipitation Assays (CHIP). They found that TO901317 caused an increased mRNA level of Short Heterodimer Partner (SHP) in HepG2 cells. In contrast, the SHP mRNA level was not affected by the oxysterols. Enhanced expression of SHP could inhibit LRH-1-mediated trans-activation of apoM promoter in HepG2 cells [11]. SHP is an inhibitory nuclear receptor activated by the FXRs that interacts physically with many nuclear receptors including LRH-1. Furthermore, it has been reported that TO901317 is a dual LXR/FXR agonist that activates FXR more efficiently than its natural ligand, the bile acids [21]. Based on the findings described above, the TO901317 downregulating apoM expression in hepatic cells may be due to the activation of the FXR/SHP pathway that inhibits LRH-1.

In the present studies, we further investigated effects of TO901317 on apoM expression in Caco-2 cells mediated via the FXR/SHP/LRH-1 pathway. TO901317 could significantly upregulate the mRNA levels of ABCA1 that is a classic LXR downstream gene [14]. LXR can bind to the HRE upstream in the proximal apoM promoter to upregulate apoM gene expression. As SHP levels were enhanced simultaneously and SHP is a classic FXR downstream gene, which suggests that FXR has been activated simultaneously [22], whereas the LRH-1 was not inhibited by the overexpressed SHP. LRH-1 was induced by the SHP and the apoM mRNA levels were upregulated. The similar phenomenon was seen in apoA1 expression [23], TO901317 could downregulate apoA1 mRNA levels in HepG2 cells but it could be upregulated in the Caco-2 cells. Furthermore we demonstrated that FXR antagonist, guggulsterone, could abolish TO901317 increased expressions of apoA1, apoM, SHP1 and LRH-1 in Caco-2 cells, although guggulsterone alone might also decrease mRNA levels of apoA1, apoM, SHP1 and LRH-1. All these findings strongly suggest that activating FXR may mediate upregulative apoM expression in the Caco-2 cells.

## Competing interests

The authors declare that they have no competing interests.

## Authors' contributions

ZCW participated in the assay of RT-PCR. JW performed the statistical analysis. YPS participated in cell culture. DD, XYZ, GHL, MBS, PNE and NX participated in the project design. All authors read and approved the final manuscript.
